# Development of a Larvicidal Nanoemulsion with *Pterodon emarginatus* Vogel Oil

**DOI:** 10.1371/journal.pone.0145835

**Published:** 2016-01-07

**Authors:** Anna E. M. F. M. Oliveira, Jonatas L. Duarte, Jesus R. R. Amado, Rodrigo A. S. Cruz, Clarice F. Rocha, Raimundo N. P. Souto, Ricardo M. A. Ferreira, Karen Santos, Edemilson C. da Conceição, Leandra A. R. de Oliveira, Alphonse Kelecom, Caio P. Fernandes, José C. T. Carvalho

**Affiliations:** 1 Laboratório de Pesquisa em Fármacos, Colegiado de Farmácia, Universidade Federal do Amapá, Campus Universitário Marco Zero do Equador, Rodovia Juscelino Kubitschek de Oliveira, KM, 02 Bairro Zerão, CEP: 68902–280, Macapá, AP, Brazil; 2 Laboratório de Nanobiotecnologia Fitofarmacêutica, Colegiado de Farmácia, Universidade Federal do Amapá, Campus Universitário Marco Zero do Equador, Rodovia Juscelino Kubitschek de Oliveira, KM, 02 Bairro Zerão, CEP: 68902–280, Macapá, AP, Brazil; 3 Laboratório de Artrópodes, Universidade Federal do Amapá, Colegiado de Ciências Biológicas, Universidade Federal do Amapá, Campus Universitário Marco Zero do Equador, Rodovia Juscelino Kubitschek de Oliveira, KM, 02 Bairro Zerão, CEP: 68902–280, Macapá, AP, Brazil; 4 Laboratório de Pesquisa, Desenvolvimento e Inovação em Bioprodutos, Universidade Federal de Goiás, Faculdade de Farmácia, Praça Universitária, 1166, Setor Leste Universitário Universitário, CEP: 74605220, Goiânia, GO, Brazil; 5 Laboratório de Produtos Naturais do Mar e de Química Bio-Orgânica, Universidade Federal Fluminense, Instituto de Biologia, Outeiro de São João Batista s/n, CEP: 24001970, Niterói, RJ, Brazil; National Centre for Cell Science, INDIA

## Abstract

*Pterodon emarginatus* Vogel is a Brazilian species that belongs to the family Fabaceae, popularly known as sucupira. Its oil has several biological activities, including potent larvicidal property against *Aedes aegypti*. This insect is the vector of dengue, a tropical disease that has been considered a critical health problem in developing countries, such as Brazil. Most of dengue control methods involve larvicidal agents suspended or diluted in water and making active lipophilic natural products available is therefore considered a technological challenge. In this context, nanoemulsions appear as viable alternatives to solve this major problem. The present study describes the development of a novel nanoemulsion with larvicidal activity against *A*. *aegypti* along with the required Hydrophile Lipophile Balance determination of this oil. It was suggested that the mechanism of action might involve reversible inhibition of acetylcholinesterase and our results also suggest that the *P*. *emarginatus* nanoemulsion is not toxic for mammals. Thus, it contributes significantly to alternative integrative practices of dengue control, as well as to develop sucupira based nanoproducts for application in aqueous media.

## Introduction

*Pterodon emarginatus* Vogel is a Brazilian species that belongs to the family Fabaceae. It is widely distributed in some regions of this country, including the states of Goiás, São Paulo and Minas Gerais [1]. *P*. *emarginatus* is commonly known as ‘sucupira’ and ‘sucupira-branca’ [2], being recognized as an important plant used in folk medicine. It was described by naturalists from 19^th^ century [3] and has been used to treat inflammation and influenza [3,4]. Essential oils mainly obtained from fruits and seeds of this species have been extensively studied. Biological activities attributed to these volatile mixtures of substances include antimicrobial [5,6], anti-ulcerogenic, anti-inflammatory [7], and cytotoxic [8] properties, besides the control of autoimmune encephalomyelitis [9]. Seeds *of P*. *emarginatus* are also used as raw material to obtain an amber-colored viscous oil that has been considered a rich source of diterpenes from the vouacapane type [10,11,12]. These terpenes are important bioactive substances, responsible for anti-inflammatory and analgesic properties [11]. In addition to the anti-inflammatory activity [2], the seed oil also present protection against oxidative stress [13] and leishmanicidal activity against promastigotes of *Leishmania amazonensis* [5]. Finally, bioguided fractionation indicated that seed oil and vouacapane diterpenes are promising natural biocontrol agents against the mosquito *Aedes aegypti* [12].

*A*. *aegypti* is the main vector of a disease called dengue, which is considered a public health problem in tropical countries. Brazil had more than 4 million reported cases of dengue between 2010–2014. Therefore, it is considered one of most important public health problems in the country [14]. Natural products from plant origin have been considered very promising for integrative practices to control vectors of tropical diseases [15,16]. Nowadays, growing interest has been devoted to the development of nano-size products for this purpose. This innovative kind of products emerged as an alternative for vector control and has potential application in different stages of insect development [17]. Merging natural products and nanotechnology opens a very promising field for integrated control programs. For example, nanoformulations may act as adulticidal [18], repellent [19] and larvicidal agents [20]. The later approach is one of most effective alternatives to prevent tropical diseases, eliminating vector’s larvae. Several natural product-based nanoformulations were reported as potential larvicidal agents [21], including nanoemulsions [20]. One major advantage of nano-size products as larvicidal products rely on the fact that they turn possible highly homogeneous dispersion of low water soluble substances in aqueous media, including herbal oils [22,23]. Moreover, natural product-based nanoformulations have being considered potential environmental low toxic agents and are thus recognized as ecofriendly products [20,21,24].

Nanoemulsions are dispersed systems constituted by two immiscible liquids often stabilized by one or more surfactants. They have small droplet size, often comprised between 20–200 nm, which allows translucent or transparent appearance [25]. Moreover, fine small droplets obtained even at relatively low surfactant concentrations generate kinetic stable systems thus preventing unstable behavior during storage of nanoemulsions, such as gravitational separation or particle aggregation [26,27]. This is a great advantage of nanoemulsions over microemulsions, which are thermodynamically stable systems that require large amounts of surfactant(s) [28]. Methods for nanoemulsion preparation are basically classified into two main groups, named high energy methods and low energy methods. High energy methods involve specific equipment, including high-shear stirring, high-pressure homogenizers and ultrasound generators. Low energy methods use physicochemical properties of the systems and often involve phase inversion [29]. It is well known that surfactants play a crucial role in nanoemulsion formation, especially if low energy methods are employed [30]. In this context, development stage for nanoemulsion preparation is a critical step in order to obtain stable fine systems. Determination of required Hydrophile Lipophile Balance (rHLB) of the oil has been recognized as an important parameter that should be investigated. This approach relies on the fact that small droplets are formed when the rHLB of oil coincides with HLB of surfactant(s) [31,32].

Despite its great biological potential, the oil of *P*. *emarginatus*, remains to our knowledge unexplored regarding the development of an innovative nanoformulation. Hence, the present study aims to obtain an optimized larvicidal nanoemulsion using *P*. *emarginatus* as the bioactive constituent.

## Materials and Methods

### Chemicals

Sorbitan monooleate and polysorbate 80 were purchased from Praid Produtos Químicos Ltda (SP, Brazil). Acetylthiocholine iodide (ATCI) and 5,5-dithiobis-2-nitrobenzoic acid (DTNB) were purchased from Sigma-Aldrich (St Louis, MO).

### Plant material

Fruits from *Pterodon emarginatus* Vogel (Fabaceae) were obtained at the Central Market of Goiânia–GO (Brazil). Identification of plant material was performed by Dr. José Realino de Paula and a voucher specimen was deposited at the Herbarium of Goiás Federal University (GO, Brazil) under the register number 41714.

### *Pterodon emarginatus* oil

Oil from *P*. *emarginatus* fruits was obtained by cold pressing, using a mini mechanical press (MPE-40 ECIRTEC). It was then weighed and hermetically stored in amber glass flask and kept at −20°C until utilization. Extraction yield (in %) was calculated according to the ratio between obtained oil and starting fruits masses.

### Identification of *P*. *emarginatus* metabolites

Identification of secondary metabolites from *P*. *emarginatus* oil was performed by comparison of the ultraviolet (UV) spectra and retention times (unpublished data) with those of external standards: β-caryophyllene (purity ≥ 98.5% Sigma–Aldrich^®^), geranylgeraniol (purity ≥ 85% Sigma–Aldrich^®^) and the vouacapan diterpenes 6α,7β-dihydroxyvouacapan-17-β-oic acid, methyl 6α,7β-di-hydroxyvouacapan-17-β-oate and 6α,hydroxyvouacapan-7β,17β-lactone (kindly provided by Dra. Dorila Piló Veloso.

### Emulsification method

Emulsification was performed using a modification of the low energy method of Ostertag et al. [27]. Emulsions were constituted as follows: 5% (w/w) of *P*. *emarginatus* oil, 5% (w/w) of surfactants and 90% (w/w) of water. Oily phase was constituted by *P*. *emarginatus* oil and surfactants, being pooled together and submitted to magnetic stirring (400 rpm) for 30 min under controlled temperature (80 ± 5°C) in a water bath. After this, the aqueous phase (distilled water) was added to the oily phase under continuous magnetic stirring (400 rpm) for 1 hour, allowing the temperature to decrease gradually to room temperature (approximately 30 min). Final step consisted in the addition of deionized water, under magnetic stirring (400 rpm, 10 min) in order to restore the original mass (10 g) of emulsions.

### Required Hydrophile-Lipophile Balance (rHLB) determination

Determination of rHLB of *P*. *emarginatus* was performed by blending sorbitan monoleate (HLB– 4.3) and polysorbate 80 (HLB– 15) at different ratios [33], in order to obtain several emulsions at a wide range of HLB (4.3–15). Composition and preparation of emulsions was according to emulsification method presented above.

### Optimized *P*. *emarginatus* nanoemulsion

An optimized *P*. *emarginatus* nanoemulsion was prepared by diluting emulsion at rHLB immediately after preparation with distilled water (1:20); final oil concentration was 2500 ppm. This nanoemulsion was immediately used for *in vivo* and *in vitro* biological assay. It was stored, protected from light, at room temperature for further analyses.

### Nanoemulsion characterization

Droplet size, polydispersity index and zeta potential of the nanoemulsion were determined by photon correlation spectroscopy (Zetasizer ZS, Malvern, UK). Emulsions from HLB determination were diluted with water (1:25, v/v), for injection [32]. Optimized *P*. *emarginatus* nanoemulsion was monitored immediately after preparation and after 1, 2, 7, 14, 21, 30 and 60 days of preparation, being diluted with distilled and deionized water (1:10, v/v). Droplet measurements were performed in triplicate and average droplet size was expressed as the mean diameter ± standard deviation.

### Larvicidal assay

*Aedes aegypti* larvae were obtained from the Arthropoda Laboratory (Universidade Federal do Amapá, Brazil). Biological assay was performed under controlled conditions, using fourth-instar larvae kept at 25±2°C, under relative humidity of 75±5% and a 12h light:dark cycle. Experimental protocol was performed according to WHO (2005) [34]. All experiments were performed in triplicate with 10 forth-instar larvae in each replicate (n = 30) Optimized *P*. *emarginatus* nanoemulsion was diluted in distilled water at 250, 100, 75, 50, 25, 12.5 ppm (relative to *P*. *emarginatus* oil). Control group was treated with deionized water. Mortality levels were recorded after 24 and 48 hours of exposure.

### Preparation of whole body homogenate

Whole body homogenate was prepared according to Sugumar et al. (2014) [23]. Larvae from treated (250 ppm) and control groups were collected and water was gently removed using tissue paper. Then each group was separately homogenized using 3.0 mL phosphate buffered saline (PBS) 0.1 M (pH = 7.5). This step was performed using a T25 Ultra-Turrax homogenizer (Ika-Werke, Staufen, Germany) running at 12000 rpm for 1 min. Then, the homogenates were centrifuged for 30 min (5000 rpm) under controlled temperature (10°C). Whole body homogenate supernatants were collected and immediately used for enzymatic assays.

### Enzymatic assays

Anticholinesterase activity was performed grossly according to the method described by Ellman et al. (1961) [35].

#### Acetylcholinesterase activity in whole body homogenate

Briefly, 0.25 mL of whole body homogenate supernatant from treated group (250 ppm) and 0.5 mL of DTNB were added to 2.0 mL of PBS. This solution was incubated for 10 min (25 ± 1°C). After this, 0.25 mL of ATCI was added and the absorbance measured at 400 nm using a UV-Mini spectrophotometer (Shimadzu). Blank was performed using whole body homogenate supernatant from control group and assays were performed in triplicate.

#### Anticholinesterase activity induced by optimized *P*. *emarginatus* nanoemulsion

Activity of acetylcholinesterase from control group whole body homogenate, after exposure to optimized *P*. *emarginatus* nanoemulsion (A_1_), was determined as follows. Solution of 0.25 mL of this nanoemulsion, 0.25 mL of whole body homogenate supernatant (control group) and 0.5 mL of DTNB to 1.75 mL of phosphate buffer was incubated for 10 min (25 ± 1°C). Then 0.25 mL of ATCI were added and the absorbance measured at 410 nm using a UV-Mini spectrophotometer (Shimadzu). Blank was obtained by replacing the ATCI by a same amount of PBS. Maximum acetylcholinesterase activity (A_2_) was achieved by replacing the amount of *P*. *emarginatus* nanoemulsion by PBS. Assays were performed in triplicate. The percentage of inhibition was calculated as follows:

$$\%\;\text{Inhibition}\;=\;100\;-\;\left[\left({\text{A}}_{1}\times \;100\right)/\;{\text{A}}_{2}\right]$$

### Acute toxicity of optimized *P*. *emarginatus* nanoemulsion on non-target species

#### Animals

This study was approved by the Animal Ethics Committee of Universidade Federal do Amapá (CEP–UNIFAP– 0018/2014). All procedures were performed according to the International Committee for animal care in accordance with established national regulations for animal experimentation. The experiments were performed using adult female Swiss albino mice (*Mus musculus*), 8 weeks age, provided by the Multidisciplinar Center for Biological Investigation in Experimental Animals Science Area from (CEMIB/SP) Campinas University. Each experimental group was composed of 3 animals. They were kept in polyethylene cages on a temperature-controlled rack (23°C ± 2°C), under a 12-hour light-dark cycle. Food and water where furnished *ad libido*, except for the 12 hours before the experiments, when food was suppressed and they had access only to water.

#### Experimental protocol

Acute toxicity studies were performed using female mice according to a modified OECD 423 protocol (2001) [36,37,38]. Treated groups had free access to optimized *P*. *emarginatus* nanoemulsion diluted in water at 250, 125 and 50 ppm (expressed as *P*. *emarginatus* oil content in aqueous media, see above) daily for fourteen days.

Behavioral analysis was performed at 0.5, 1, 2, 4, 8, 12 and 24 h after the oral treatment and then daily for fourteen days. Behavioral changes (hyperactivity, convulsions, vocal fremitus, irritation, stereotyped movements, touch response, salivation, tremors, writhing, body distension, ptose, sleepiness, defecation, diarrhea, piloerection), weight, food and water intake, clinical signs of toxicity and mortality were recorded daily. At the end of fourteen days, they were sacrificed by cervical dislocation and taken to autopsy for macroscopic observation of the organs (heart, lung, liver, kidney and spleen).

#### Statistical analysis

Analysis of variance (ANOVA) followed by Tukey´s test was conducted using the Software GraphPad Prism v.5.03 (San Diego, California, USA). Differences were considered significant when P≤ 0.05. Probit analysis was performed with 95% confidence interval for LC_50_ determination.

## Results and Discussion

**Fig 1:**
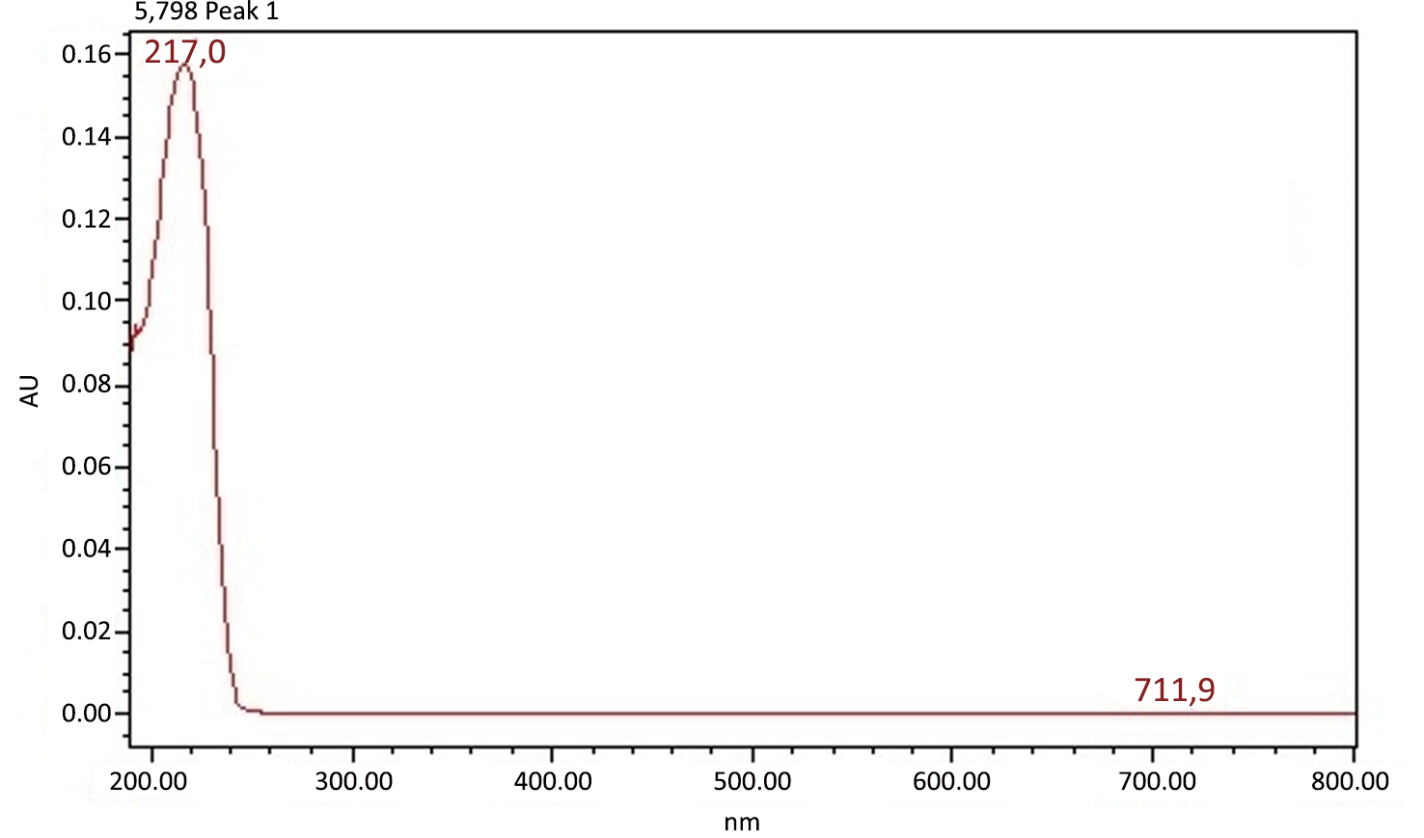
UV spectra obtained by HPLC-DAD of diterpene MHV.

**Fig 2:**
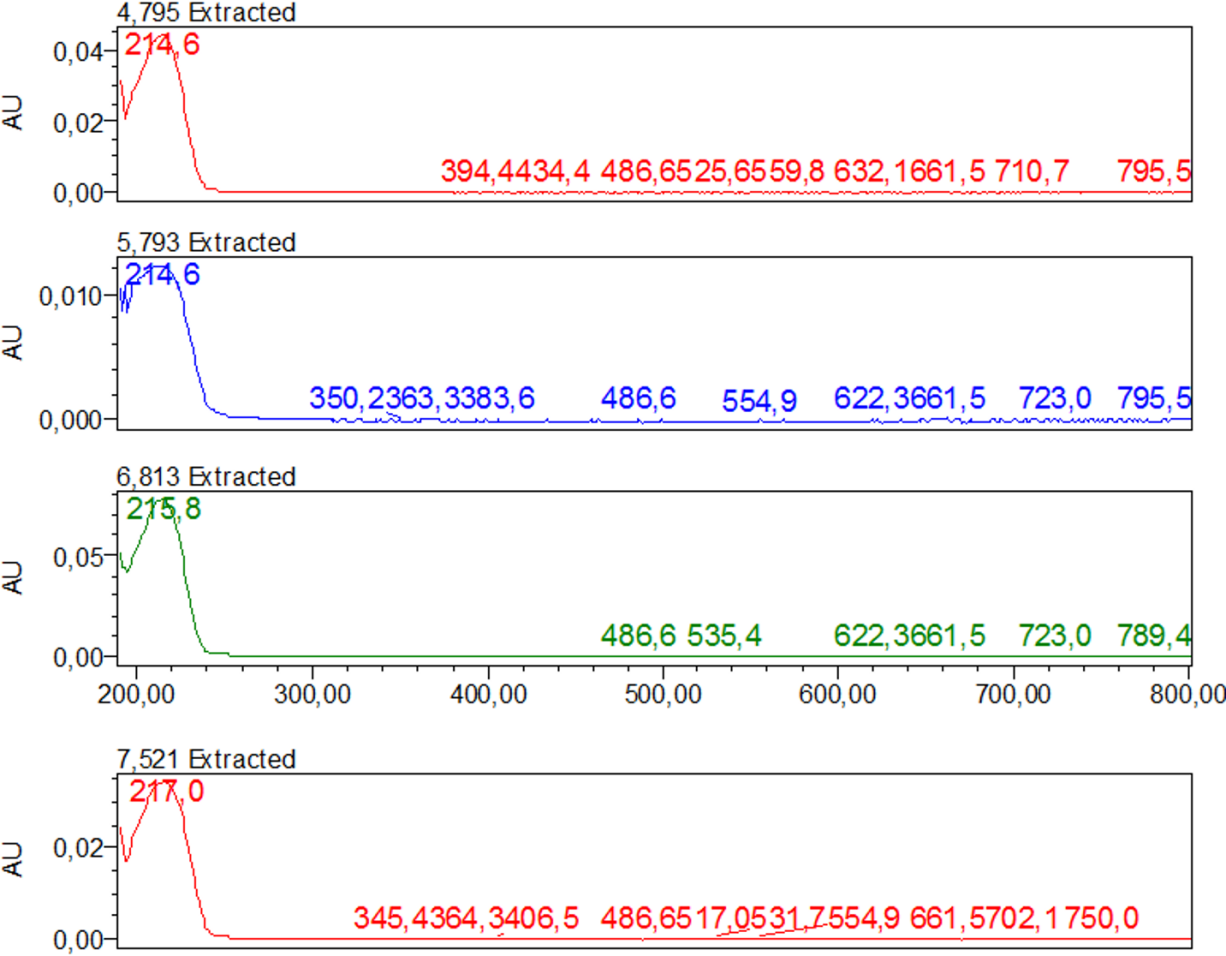
UV spectra from non-identified compounds from *P*. *emarginatus* oil suggesting presence of other vouacapan diterpenes.

Extraction of *P*. *emarginatus* fruits yielded 30% (w/w) of a viscous yellow oil. Diterpenes methyl 6α,7β-dihydroxyvouacapan-17-β-oate (MHV), geranylgeraniol and sesquiterpene β-caryophyllene were detected on *P*. *emarginatus* oil by comparison to external standards. Vouacapan diterpenes have been considered potential bioactive substances from fruits of *P*. *emarginatus* [11]. Moreover, literature data indicates that some of them, including methyl 6α,7β-dihydroxyvouacapan-17-β-oate, presented potential larvicidal activity [12]. Fig 1 shows the UV spectrum of the diterpene MHV. The UV spectra recorded for other substances present in chromatogram of *P*. *emarginatus* oil that may be associated to additional vouacapan skeleton diterpenes in this sample are presented in Fig 2.

Natural products display a wide range of biological activities that make them promising candidates for a number of applications. Among the various classes of natural products, terpenoids and more specifically diterpenes are potentially useful as insecticide agents [39]. However, these substances often possess poor water solubility, being usually much more soluble in toxic organic solvents. Alternatives to enhance their water solubility are desirable looking for viable eco-friendly active products [40]. Classical chemical reactions may be used, giving rise to water soluble compounds such as forming terpenoid salts [41]. Another strategy relies on the development of stable nanosystems, which can improve water solubility of these substances by entrapping then into micelles with nano-size diameter [42]. Nanobiotechnology is an emerging area with great potential for innovative products, including eco-friendly pesticides. Thus, we decided to develop *P*. *emarginatus* oil in water nanoemulsions, using oil as the internal phase in order to enhance its water solubility and verify its potential as a vector control.

Emulsions at HLB 4.3, 5, 6, 7, 8 and 9 presented some signals of instability after preparation, including presence of sedimentation and phase separation. Emulsions at HLB 10, 11, 12, 13, 14 and 15 presented slight yellow appearance and homogeneous aspect. After one day of storage, emulsions at HLB 15 and 14 presented highest mean droplet size (HLB 15: 459.2 ± 6.8 nm; HLB 14: 262.1 ± 4.3 nm) and polydispersity index (HLB 15: 0.393 ± 0.006; HLB 14: 0.192 ± 0.008). Emulsions at HLB 10 and 13 presented mean droplet size below 200 nm after 1 day of preparation. However, after 7 days, values increased above this upper limit. Smallest droplets size was observed for emulsions at HLB 12 (126.7 ± 0.9 nm) and HLB 11 (135.8 ± 0.2 nm), analyzed after 1 day.

After 7 days, smallest mean droplet size was observed for formulation at HLB 11. Moreover, this emulsion presented less variation of both mean droplet size and polydispersity index parameters, when compared to emulsion at HLB 12. Characterization of nanoemulsions must involve determination of droplet size, since these nanodispersed system are classified according to droplet diameter. Dynamic light scattering (DLS) is one of most used technique for this purpose, giving average size and size distribution of droplets, also offering some advantages, such as fast and easy analysis. However, complementary techniques may be used to corroborate data obtained by DLS, such as transmission electron microscopy, which measures mean diameter of individual droplets and also provides important information about surface and morphology of droplets [43]. Zeta potential is also considered an important parameter regarding stability of nanoemulsions. It is associated to surface potential of droplets that constitute these dispersed systems. Zeta potential values obtained in the present study were in accordance with stable behavior [44]. Mean droplet size, polydispersity index and zeta potential of emulsions at HLB 11 and 12 are presented in Table 1.

**Table 1:** Mean droplet size, polydispersity index and zeta potential during of emulsions prepared during rHLB determination of *P*. *emarginatus* oil.

	1 Day			7 Days		
HLB	Mean droplet size (nm)	Polydispersity index	Zeta potential (mV)	Mean droplet size (nm)	Polydispersity index	Zeta potential (mV)
11	135.8 ± 0.2	0.173 ± 0.002	-27.2 ± 0.6	160.3± 1.4	0.188± 0.02	-24.3 ± 0.5
12	126.7 ± 0.9	0.096 ± 0.019	-25.1 ± 1.3	182.0± 0.7	0.140± 0.005	-25.6 ± 1.1

Hydrophile-Lipophile Balance was formerly described as an useful scale for selecting emulsifiers. Low HLB values were attributed to more lipophilic agents and high HLB values were attributed to more hydrophilic agents [45]. It is well established that most stable emulsion, and therefore smaller droplets, are achieved when HLB of surfactant(s) coincides with rHLB of the oil [46]. In this context, it is desirable that rHLB of oily phase constituents, including herbal oils, should be measured. Basically, the procedure aiming this purpose has been performed by blending pairs of surfactants at different ratios and monitoring most stable emulsion as a valuable tool to determine rHLB [32,47]. Thus, our results suggest that oil from fruits of *P*. *emarginatus* has HLB of 11. Some of our recent studies reported generation of nanoemulsions with good physical properties using this approach [33,48,49,50], as it could be achieved in the present study. However, in some cases it could be observed that it was not possible to obtain nanoemulsions during rHLB determination [46,47]. To overcome this problem, some studies with important Brazilian herbal oils were carried out with organic solvent-based methods, high energy methods or merging both techniques. Addition of aqueous phase to an oily phase containing copaiba (*Copaifera multijuga*) oil was unsuccessful to induce formation of nanoemulsions, being required an additional step using a high pressure homogenizer to generate intense disruptive forces and achieve droplets with mean diameter around 160–300 nm. In this same study, spontaneous generation of nanoemulsions was only achieved by solubilizing oily phase with organic solvents, resulting in mean droplet size around 160–315 nm [51]. In another study carried out with andiroba (*Carapa guianensis*) and aroeira (*Schinus molle*) oils, solubilization of oily phase with organic solvent allowed only to obtain coarse emulsions; nanoemulsions (respectively with mean droplet around 240 and 130 nm) could finally be produced by a further high pressure homogenization step [52]. Besides the aforementioned disadvantage of high energy methods due to increasing costs of the process, utilization of organic solvents should be avoided if development of an eco-friendly product is desired. It is well known that a supplementary evaporation step using a rotary evaporator should be performed to remove the organic solvent from internal or external phases of the nanoemulsion. However, literature data suggests that residual levels of the organic solvent may remain entrapped and gradually released [53].

**Fig 3:**
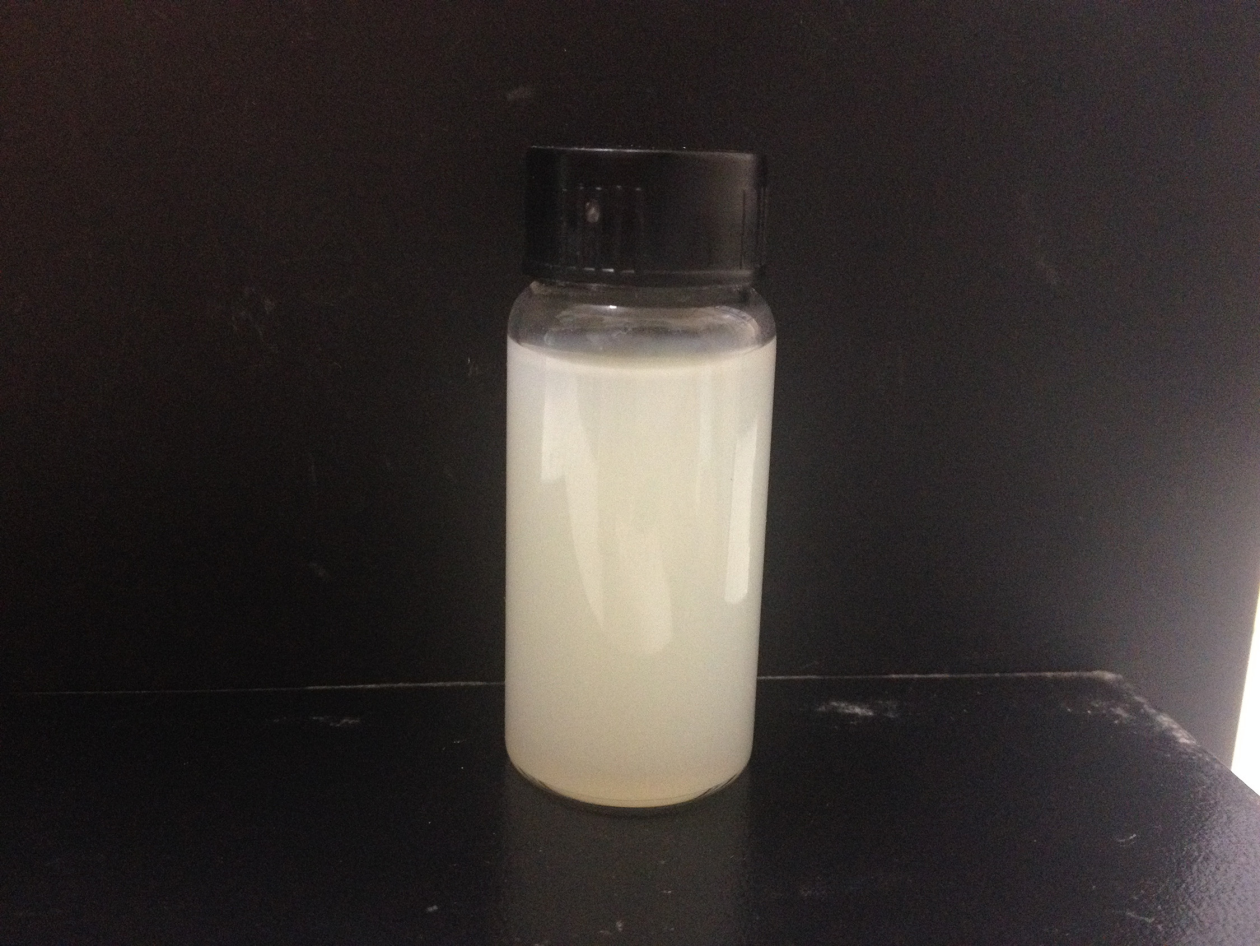
Optimized *P*. *emarginatus* nanoemulsion used for in vitro and in vivo biological assays.

**Fig 4:**
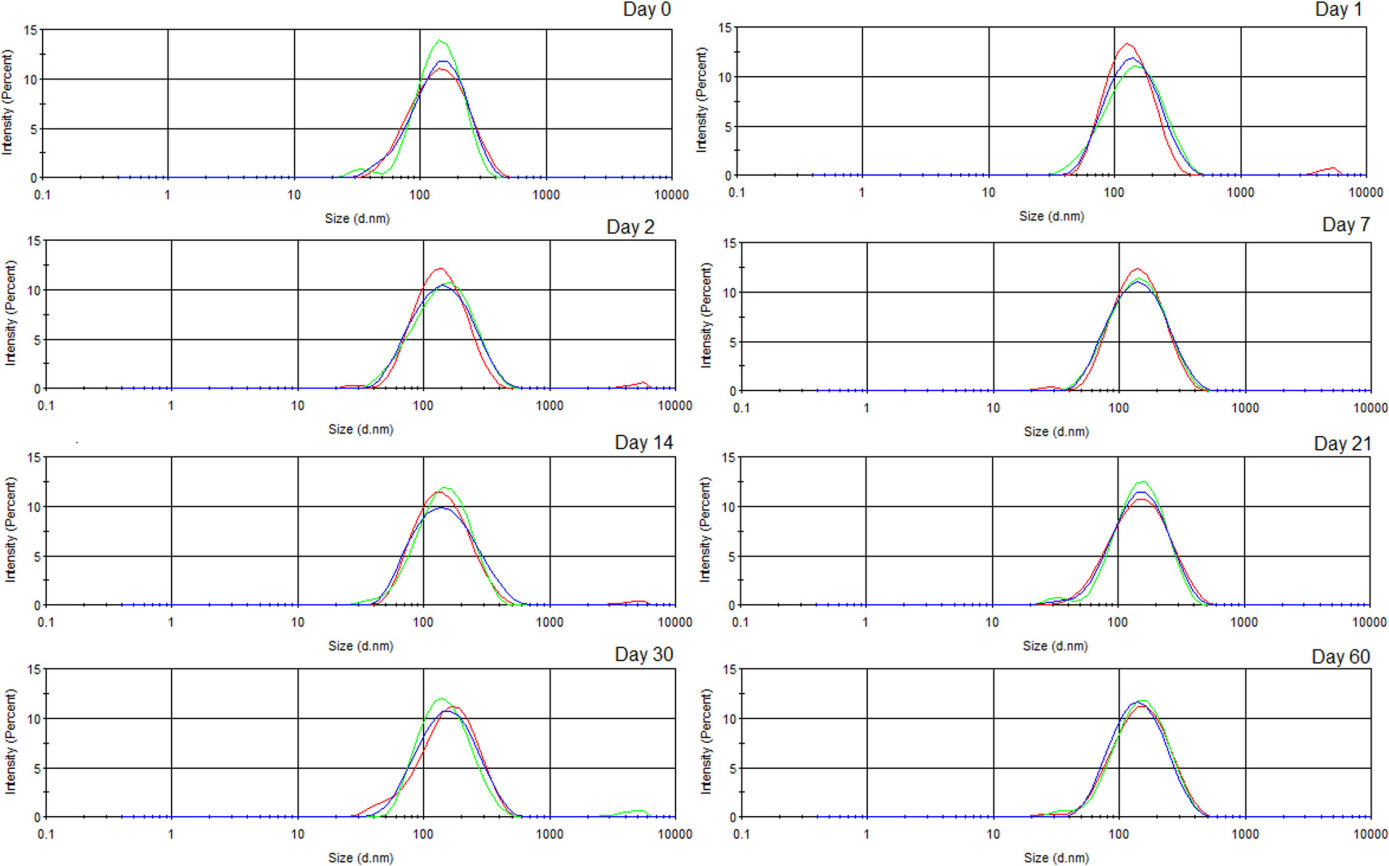
Mean droplet size—Day 0: 125.1 ± 0.5 nm; Day 1: 124.4 ± 0.4 nm; Day 2: 125.6 ± 0.5 nm; Day 7: 124.8 ± 0.3 nm; Day 14: 127.5 ± 0.2 nm; Day 21: 131.0 ± 0.5 nm; Day 30: 134.3 ± 0.8 nm; Day 60: 129.2 ± 1.0 nm. Polydispersity index—Day 0: 0.175 ± 0.014; Day 1: 0.185 ± 0.012; Day 2: 0.196 ± 0.006; Day 7: 0.174 ± 0.003; Day 14: 0.194 ± 0.013; Day 21: 0.192 ± 0.007; Day 30: 0.210 ± 0.006; Day 60: 0.193 ± 0.001.Zeta potential—Day 0: -30.9 ± 0.4 mV; Day 1: -29.6 ± 1.4 mV; Day 2: -29.6 ± 1.4 mV; Day 7: -33.1 ± 3.3 mV; Day 14: -35.4 ± 2.8 mV; Day 21: -35.4 ± 1.2 mV; Day 30: -47.1 ± 1.5 mV; Day 60: -41.0 ± 6.34 mV.

Due to their small size and potential kinetic stability, even at relatively low surfactant concentrations [25], nanoemulsions have been considered potential innovative products for a wide range of applications. Transparent or translucent aspect of nanoemulsions is also an attractive, being this type of nanoformulation very useful to incorporate lipophilic agents, such as herbal oils, into aqueous products [54]. Special attention is given for natural-products-based nanoemulsions as ecofriendly pesticides, considering their potential advantages, including low risk for non-target organisms and the fact that they are often more easily biodegraded [20,23,42]. Optimized *P*. *emarginatus* nanoemulsion was prepared by diluting nanoemulsion at HLB 11 and presented fine appearance and translucent aspect (Fig 3). It was also characterized as an O/W system, being able to be dispersed in aqueous media. Almost no variation was observed for droplet size and polydispersity index during storage (60 days), suggesting that the system has a great tendency to achieve kinetic stability. Narrow size distribution with monomodal aspect was observed, being in accordance with high homogeneity and low polydispersity nanodispersed systems [55,56]. Particle size distribution during storage of optimized *P*. *emarginatus* nanoemulsion is presented in Fig 4.

As part of our ongoing studies on the development of larvicidal nanoemulsions with Brazilian herbal oils, we decided to investigate the effects of *P*. *emarginatus* nanoemulsion on *Aedes aegypti* larvae. Groups of larvae treated with optimized *P*. *emarginatus* nanoemulsion at 250 ppm (expressed as *P*. *emarginatus* oil content in aqueous media) presented mortality level of 100%. According to literature data, a natural product is considered a promising larvicidal agent when mortality of treated group at 250 ppm reaches mortality levels higher than 75% [57]. A similar effect was observed for a larvicidal nanoemulsion against *Culex quinquefasciatus*, which induce 98% of mortality at 250 ppm after 24 h of treatment [23]. No mortality was observed in the *A*. *aegypti* control groups. ANOVA test indicated statistical differences in mortality between tested concentrations after 24 h (F value = 27.88; p = 0.0000) and 48 h (F value = 31.10; p = 0.0000). It was also observed that mortality rate was dependent on time for treated groups at 12.5, 25, 50 and 75 ppm. This observation is in accordance with previous data of larvicidal nanoemulsions, in which mortality levels increased with time exposure [58]. Mortality levels induced by nanoemulsion prepared with *P*. *emarginatus* oil are presented in Table 2. LC_50_ after 48 h was estimated as 34.75 (7.31–51.86) ppm (expressed as *P*. *emarginatus* oil content in aqueous media).

**Table 2:** Mortality levels (%) of *Aedes aegypti* larvae after treatment with optimized *P*. *emarginatus* nanoemulsion (expressed as *P*. *emarginatus* oil content in aqueous media).

		Tested concentrations					
Exposure time (h)	Control	12.5 ppm^*^	25 ppm^*^	50 ppm^*^	75 ppm^*^	100 ppm	250 ppm
24	0	0	0	40 ± 17.32^a^	50 ± 20.0^a^	90 ± 0.0^c^	100 ± 0.0
48	0	23.33 ± 5.77^a^	50 ± 17.32^b^	63.33 ± 5.77^b^	83.33 ± 5.77^c^	93.33 ± 5.77^c^	100 ± 0.0

Data is expressed as mean ± standard deviation

Analysis of variance (ANOVA) followed by Tukey´s test was performed to evaluate statistical significance of the results (n = 30). Experiment was performed in triplicate and each replicate was composed by 10 larvae.

^*^ Statistical significant increase in mortality level as function of exposure period

Means in the same line with different superscript mean statistical significant difference (P < 0.05)

Most studies carried out with larvicidal nanoemulsions do not present LC_50_ values [20,23,48,50]. Anjali et al. (2012) [58], in turn, observed that neem nanoemulsion with mean droplet size of 93.0 nm presented LC50 of 25.99 ppm against *C*. *quinquefasciatus*. Regarding nanoparticles, a study aiming the investigation of larvicidal potential of phyto-based nickel nanoparticles indicated a LC_50_ of 534.83 ppm against *A*. *aegypti*, while temephos showed a LC_50_ of 507.62 ppm [59]. Considering that bioaccumulation is considered a major problem regarding environmental toxicity [60], safety of this metal-based nanoparticles should be deeply investigated. In this respect, our results suggest that optimized *P*. *emarginatus* nanoemulsion presented a larvicidal activity in accordance with literature data for classical herbal derived nanoemulsions. It also may be considered potentially more effective than other nanoformulations and classical chemicals used for vector control.

Despite aforementioned advantages of oil in water nanoemulsion as larvicidal agents, a possible increase of biological activity should also be mentioned. Study carried out with nanoformulations containing herbal oil and geraniol indicated a positive correlation between larvicidal and small size droplets [22]. Anjali et al. (2012) [58] also reported that neem nanoemulsions with smaller mean droplet size presented smaller LC_50_ values against *C*. *quinquefasciatus*. Hence, development of a nanoemulsion can be considered a good approach to develop viable innovative products for insect control, even enhancing biological activity of natural compounds. Classical bioactive Brazilian herbal oils, such as *Carapa guianensis* and *Copaifera sp* presented LC_50_ around 50 and 150 ppm, respectively [61]. On the other hand, previous study carried out with vouacapan diterpenes from *P*. *emarginatus* indicated that MHV presented LC_50_ of 21.76 ppm [12]. In the present study, the observed LC_50_ is closer to those expected for isolated diterpenes and indicated that this nanoformulation is more active than some non-formulated herbal oils of great interest.

Several natural products from plant origin were reported as potential insecticides, including diterpenes [62]. One of the possible mechanisms involved is inhibition of acetylcholinesterase of the insects [39,63]. Comparison between acetycholinesterase activity of *A*. *aegypti* larval homogenates (control and tested group) indicated no statistically significant difference (P>0.05) regarding acetylcholinesterase activity. However, it was observed that *P*. *emarginatus* oil-based nanoemulsion added to whole body homogenate from control group of *A*. *aegypti* larvae presented anticholinesterase activity, being able to inhibit the enzyme activity around 50.22%. Previous study performed with terpenoids indicated that some of them induced a reversible inhibition of acetylcholinesterase, verified by recovery of enzyme activity after 15 min of incubation [63]. Thus, it could be suggested that acetylcholinesterase inhibitors from *P*. *emarginatus* detached from the enzyme, explaining similar acetylcholinesterase activity on *A*. *aegypti* whole body larval homogenates (control and treated groups).

*A*. *aegypti* preference for clearwater and its ability to grow and develop in human-made receptacles, such as discarded pots, tires and water storage containers is well known [64]. Moreover, this vector became fully adapted to human environment [65]. Vector control in drinking water sources and containers using larvicidal chemicals, such as temephos, is recognized as an effective strategy [66]. However, utilization of home pesticides may generate intoxication of human [16] and/or domestic animals, such as dogs and cats [67]. Thus, low toxicity for both humans and other non-target organisms is a main characteristic that should be considered for development of novel larvicidal products [68].

**Fig 5:**
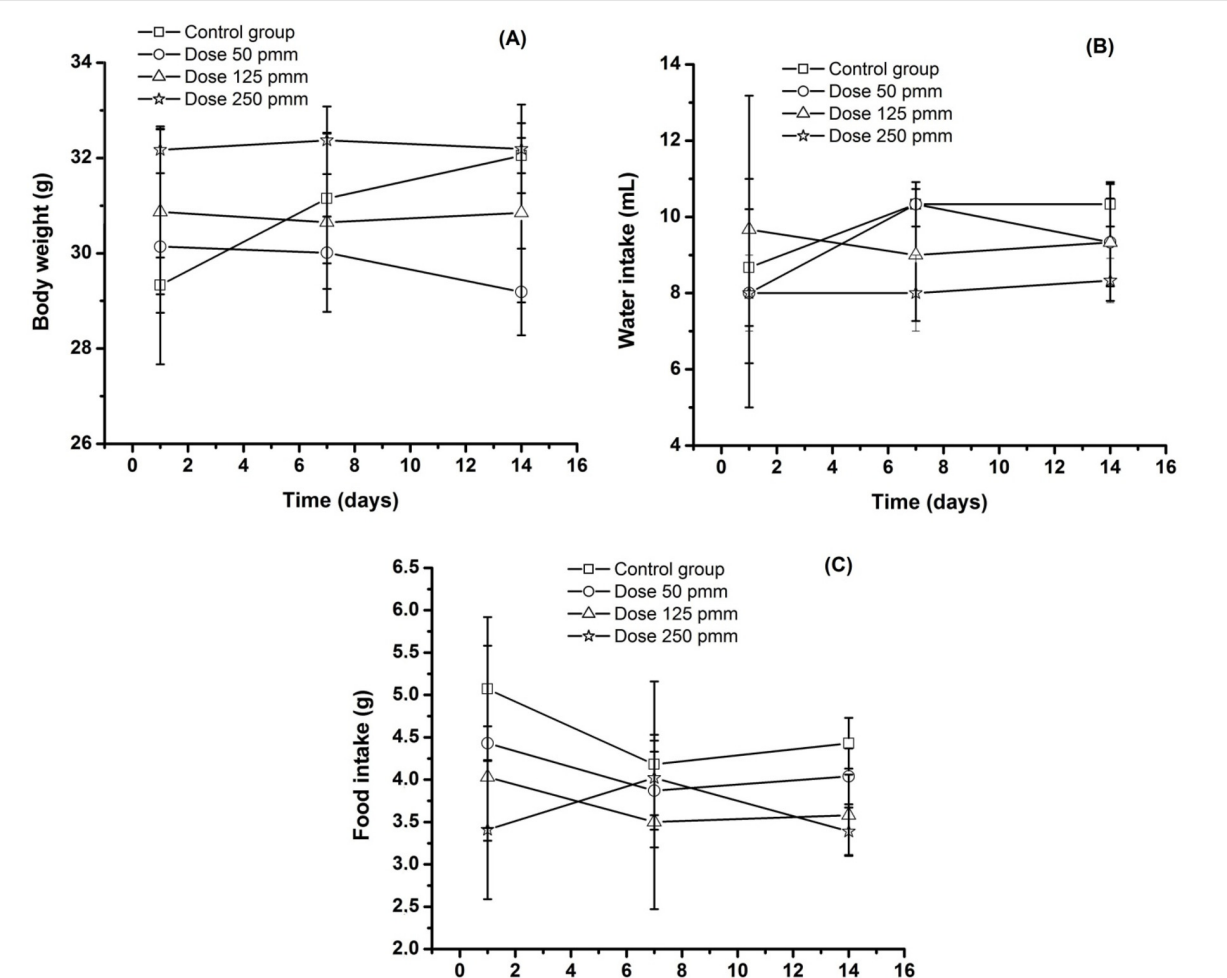
Analysis of body weight (A), water (B) and food intake (C) variation in mice treated with optimized *P*. *emarginatus* nanoemulsion.

In the present study, it was decided to investigate the action of optimized *P*. *emarginatus* nanoemulsion on non-target species using adult female Swiss albino mice (*Mus musculus*) as a model for mammals. No behavioral alteration, death or macroscopical changes in organs (heart, lung, liver, kidney and spleen) could be observed (Data not shown). Moreover, the absence of any significant change in body weight, food and water intake (Fig 5) also suggests that optimized *P*. *emarginatus* nanoemulsion may be not toxic for mammals.

## Conclusions

Oil from seeds of *P*. *emarginatus* is recognized as a plant derived product with great biological potential, including for larval control of dengue vectors. However, it remained unexplored until this moment regarding development of a nanobiotechnology product. The present study allowed development of a novel nanoemulsion with larvicidal activity against *A*. *aegypti* along with determination of rHLB of this oil. Moreover, it could be suggested that the mechanism of action may involve reversible inhibition of acetylcholinesterase and that the nanoemulsion may be safe for mammals.

It is worth mentioning that our optimized *P*. *emarginatus* nanoemusion was prepared by a low energy and solvent-free method, reducing costs of the process and being in agreement with eco-friendly products requirements. Absence of coating which is necessary for nanoparticle preparation with no impairment to physical stability and larvicidal activity can be considered an advantage in terms of process costs. Moreover, utilization of eco-friendly surfactants is also an advantage, avoiding bioaccumulation which is associated to other type of nanoformulations. Thus, it contributes significantly to alternative integrative practices of dengue control, as well as developing of sucupira based nanoproducts for application on aqueous media.
